# Lasting the distance: The survival of alien birds shipped to New Zealand in the 19th century

**DOI:** 10.1002/ece3.6143

**Published:** 2020-03-07

**Authors:** Pavel Pipek, Tim M. Blackburn, Steven Delean, Phillip Cassey, Çağan H. Şekercioğlu, Petr Pyšek

**Affiliations:** ^1^ Department of Invasion Ecology Czech Academy of Sciences Institute of Botany Průhonice Czech Republic; ^2^ Department of Ecology Faculty of Science Charles University Prague Czech Republic; ^3^ Centre for Biodiversity and Environment Research University College London London UK; ^4^ Institute of Zoology Zoological Society of London London UK; ^5^ Centre for Applied Conservation Science School of Biological Sciences The University of Adelaide Adelaide SA Australia; ^6^ Department of Biology University of Utah Salt Lake City UT USA; ^7^ College of Sciences Koç University Istanbul Turkey; ^8^ Conservation Science Group Department of Zoology University of Cambridge Cambridge UK

**Keywords:** 19th century, alien birds, invasions, New Zealand, shipping, survival, transport

## Abstract

Invasive alien species are a major threat to biodiversity and human activities, providing a strong incentive to understand the processes by which alien invasion occurs. While it is important to understand the determinants of success at each of several invasion stages—transport, introduction, establishment, and spread—few studies have explored the first of these stages. Here, we quantify and analyze variation in the success of individual animals in surviving the transport stage, based on shipping records of European passerines destined for New Zealand. We mined the original documents of Acclimatisation Societies, established in New Zealand for the purpose of introducing supposedly beneficial alien species, in combination with recently digitized newspaper archives, to produce a unique dataset of 122 ships that carried passerines from Europe to New Zealand between 1850 and 1885. For 37 of these shipments, data on the survival of individual species were available. Using generalized linear mixed models, we explored how survival was related to characteristics of the shipments and the species. We show that species differed greatly in their survival, but none of the tested traits accounted for these differences. Yet, survival increased over time, which mirrors the switch from early haphazard shipments to larger organized shipments. Our results imply that it was the quality of care received by the birds that most affected success at this stage of the invasion process.

## Graphical abstract

While it is important to understand the determinants of success at each of several invasion stages—transport, introduction, establishment, and spread—few studies have explored the first of these stages. Here, we quantify and analyse variation in the success of individual animals in surviving the transport stage, based on shipping records of European passerines destined for New Zealand. Our results imply that it was the quality of care received by the birds that most affected success at this stage of the invasion process.

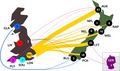

## 1 INTRODUCTION

The transportation and subsequent introduction of species to regions beyond their natural distribution, where they do not naturally occur (here termed aliens), is one of the principal ways in which human activities are changing the natural world (e.g., Bellard, Genovesi, & Jeschke, 2016; Blackburn et al., 2011; Clavero & Garcia‐Berthou, 2005; Lockwood & McKinney, 2001). Typically, the first records of alien species are reported after they have established and when individuals are observed in a novel environment. This is particularly the case in recent decades, as most extant alien incursions are accidental introductions (Dyer, Redding, & Blackburn, 2017; Seebens et al., 2017). However, these introductions will be a subset of all the individuals and species that have been transported (Lonsdale, 1999), as some will not survive the trip, and some of those surviving will not subsequently be liberated (for the latter case, see Pipek, Pyšek, & Blackburn, 2015). This makes it difficult to assess how the transport stage influences the invasion process. For example, without a well‐defined pool of species that were selected for transportation, we may well have a biased impression about which species traits were desirable or made the birds more prone to being transported. Conversely, the transported individuals might have a skewed sex ratio (and therefore reduced effective population size) or be delivered in such poor condition that their subsequent chances of survival would be low.

Despite its relevance to the invasion process, the transport stage itself has rarely been analyzed (Duncan, Blackburn, & Sol, 2003). For birds, as far as we are aware, only two studies have compared species that have been transported versus those that have not, using data on the composition of species in trade (Cassey, Blackburn, Russell, Jones, & Lockwood, 2004; Su, Cassey, & Blackburn, 2014). To date, no studies have systematically analyzed the causes of variation in the success of individual animals in surviving the transport stage. Here, we take advantage of a large experiment in nature—Acclimatisation Society introductions of alien species to New Zealand—to perform such an analysis for the first time.

In the mid‐19th century, the colonization of many parts of the world by Europeans was associated with attempts to naturalize alien species considered to be beneficial in the new colonies or back in Europe (di Castri, 1989; Lever, 1992). Acclimatisation Societies were founded with the specific aim of promoting the naturalization of species (Lever, 1992), mainly from Britain to its colonies (Long, 1981). The several larger Societies founded in New Zealand were a particularly rich source of information on the invasion process for alien birds in New Zealand (McDowall, 1994). Notably, they maintained detailed records of which species they introduced and which introductions resulted in established populations, and summaries of these records were subsequently collated and published in catalogue form (Drummond, 1906; Thomson, 1922). These catalogues have been the basis of a suite of analyses of patterns of introduction, establishment, and spread in alien birds in New Zealand (reviewed in Duncan, Blackburn, & Cassey, 2006). However, the records of the Acclimatisation Societies and other resources include more information than was included in the catalogues, notably on the composition of specific shipments of bird species to New Zealand destined for use by the Societies (Pipek et al., 2015). Much of this information was also duplicated in local newspapers, now readily available in searchable online archives (Pipek et al., 2015; Star, 2014). In fact, some data are stored only in the newspapers, as shipments of birds commenced before Acclimatisation Societies were founded, some Society records were lost, and some shipments were private endeavors. These sources allowed us to collate a unique dataset of ships for which there was reliable information both about the number of individual birds loaded at the ports of origin and the number that survived the journey to New Zealand. We use these data to document survival rates over the transport stage for individual birds of numerous alien species and to test hypotheses for variation in these rates.

Specifically, we use the records for sailing ships that transported birds from Europe to New Zealand in the period 1850–1885, when birds were most intensively shipped, to test whether survival through the transport stage of these invasions related to characteristics of the voyage (event‐level) or of the species (Peoples & Goforth, 2017) shipped. We restricted our analyses taxonomically, to the order of passerine birds: These species were selected for transport largely with the same aim, to control insect pests, and were intended for direct release. We first tested whether survival rates differed by shipment and by species shipped and then tested for causes of the variation observed. For shipments, we tested whether variation in survival related to (a) the duration of the voyage; (b) the year; and (c) the season of the year when the shipment departed from Europe. We hypothesized that (a) longer trips would result in higher mortality due to more opportunities for unwelcome events (accidents, epidemics, and food shortage, see Table S1). We assumed that (b) Acclimatisation Societies might gain experience and improve their skills in transporting birds over time. We also assumed that (c) season was important, for example, because birds taken during (e.g., Hawke's Bay Herald, 1865) or just after breeding might be exhausted and/or stressed, or those taken in Northern Hemisphere's spring might have difficulties coping with the Southern Hemisphere winter, for example, for not having remoulted first—remoulting takes about the same time as the transport itself and normally would begin in late summer (Morrison et al., 2015), while traveling through warmer climates might encourage the birds to shed even more feathers (Kiat, Vortman, & Sapir, 2019). Unnatural moult in the Tropics followed by colder temperatures in higher latitudes was reported as the cause of death of many small birds on the otherwise successful shipment by the Charlotte Gladstone in 1872 (Farr, 1872).

For the species shipped, we tested whether any variation in their survival was related to (a) sexual dimorphism; (b) migratory behavior; (c) diet type; and (d) body size. We focused on these traits based on previous research. For example, larger‐bodied species generally have lower mortality rates, which may affect their ability to survive periods of cold or starvation on long voyages (Peters, 1983), while food for granivorous species may be easier to provision. Sexual dimorphism and migratory behavior are reported to correlate negatively with establishment success in birds (Cassey et al., 2004; Sorci, Moller, & Clobert, 1998) and could possibly negatively affect survival during transport too, for example, due to higher mortality of males in sexually dimorphic species (Promislow, Montgomerie, & Martin, 1992) or migratory restlessness in the case of migrants.

## 2 METHODS

### 2.1 Data collection

Data on shipments of European passerines were extracted from the documents of the Acclimatisation Societies (published or unpublished, obtained from New Zealand libraries) and digitized newspaper archives. The former included annual reports and “Minutes” books with the transcripts of meetings, correspondence between the Societies and their agents in London, and Society Cashbooks. The digitalized newspaper articles came mainly from the New Zealand archive Paperspast (https://paperspast.natlib.govt.nz; see more details in (Pipek et al., 2015)), but partly also from the Australian (https://trove.nla.gov.au) and British (https://www.britishnewspaperarchive.co.uk) archives.

To locate relevant articles from the newspaper archives, we searched for all articles containing the common names of bird species known to be transported to New Zealand or to Acclimatisation Societies (ca. 200,000 articles in total). For some species, this required using queries for a different name than is used nowadays (e.g., dunnocks *Prunella modularis* were hedge sparrows, greenfinches *Chloris chloris* were sometimes referred to as green linnets, and robins *Erithacus rubecula* as redbreasts, see Table S3). We tried both singular and plural forms. When the common name (or the synonym) was composed of two words, all combinations (i.e., with space or dash as a separator or without any gap) were used. Following Pipek, Blackburn, and Pyšek (2019), whenever larks and thrushes were referred to, we assumed it concerned skylarks *Alauda arvensis* and song thrushes *Turdus philomelos*, unless it was explicitly stated that these were mistle thrushes *Turdus viscivorus* or woodlarks *Lullula arborea*. Most articles located were unrelated to the bird species of interest (e.g., the name referred to a person, horse or ship), were copies of other articles, or were identified due to text having been incorrectly processed in the digitization process (e.g., rock or book instead of rook *Corvus frugilegus*). To eliminate these irrelevant articles and also to avoid reading articles concerning multiple species several times, the extracts of the articles were first saved locally in an SQL database. The extracts helped us then to filter unwanted articles in a way mimicking “the sieve of Eratosthenes” method—each time we discovered during reading the articles a new reason for exclusion (e.g., robin was in fact “Mr. Robin” or “X. Y. Robin”)—we marked similar articles in the database using regular expressions in PHP, and these were then removed from the list of articles to read. After elimination of the unwanted papers, we were left with 1,053 articles that related to shipments of passerines, concerning 122 shipments.

For analysis, we used only those shipments and species for which there was information on survival rate, that is, the number of individuals loaded and surviving the journey. We also excluded data that were doubtful in terms of taxonomy or numbers of birds transported. In some cases, the numbers were reported in taxonomic pairs; in particular, blackbirds *Turdus merula* and thrushes *T. philomelos,* and yellowhammers *Emberiza citrinella* and chaffinches *Fringilla coelebs* were often lumped together in reports. As a result, for some shipments, not all of the taxa that were on board were included in the analysis. From the remaining shipments, we excluded steamers (*n* = 4), as these had either a different route from the U.K. to New Zealand—the species first travelled to Panama, where they had to be reshipped—or their trip was much shorter in duration and they would thus be substantial outliers. In total, we identified 528 shipment × taxon combinations, of which 144 (listed in Table S1) were suitable for the analysis, comprising 37 shipments and 23 species.

For each species, data on body mass (g), diet (plant/seed, invertebrate, and omnivore), and binary classifications as sexually dimorphic, sedentary, facultative migrant, short‐distance migrant, and long‐distance migrant, were extracted from Storchová and Hořák (2018) and a global database containing the ecological traits of all of the world's bird species (Şekercioğlu et al., 2019), see Table S2. The data regarding length of the voyage and departure/arrival ports were extracted from http://freepages.rootsweb.com/~shipstonz/genealogy/shipstonz.html, except those easily obtainable directly from the articles.

### 2.2 Statistical analysis

We calculated survival as the proportion of individuals of each species that survived each voyage, out of the total number shipped. We then examined the relationship between survival and characteristics of the voyage for each shipment, including the duration (in days), season of departure from Europe, and year of arrival of the voyage in New Zealand using generalized linear mixed models. A beta‐binomial variance function (with logit link) was used to account for extrabinomial variation in the grouped survival counts. Variations in survival among bird species, and among ship voyages, were accounted for as random intercept effects in the analysis, and these relationships were visualized by plotting the conditional modes of the factor levels of each random intercept term. In some models, natural splines were used to assess evidence for nonlinearities in the relationships between survival and both the duration and year of the voyage. Likelihood ratio tests were used to examine evidence for relationships between survival and the fixed effects. Models were fitted using the glmmADMB package (Skaug, Fournier, Bolker, Magnusson, & Nielsen, 2016) in the R software environment (version 3.4.4) for statistical and graphical computing (R Core Team, 2018). We examined whether variation in survival was associated with body mass, diet, dimorphism, or different migratory strategies, by including these traits as additional explanatory variables in the generalized linear mixed models described above. Likelihood ratio tests were used to examine evidence for relationships between survival and the individual traits.

## 3 RESULTS

### 3.1 Ships and taxa

**Figure 1:**
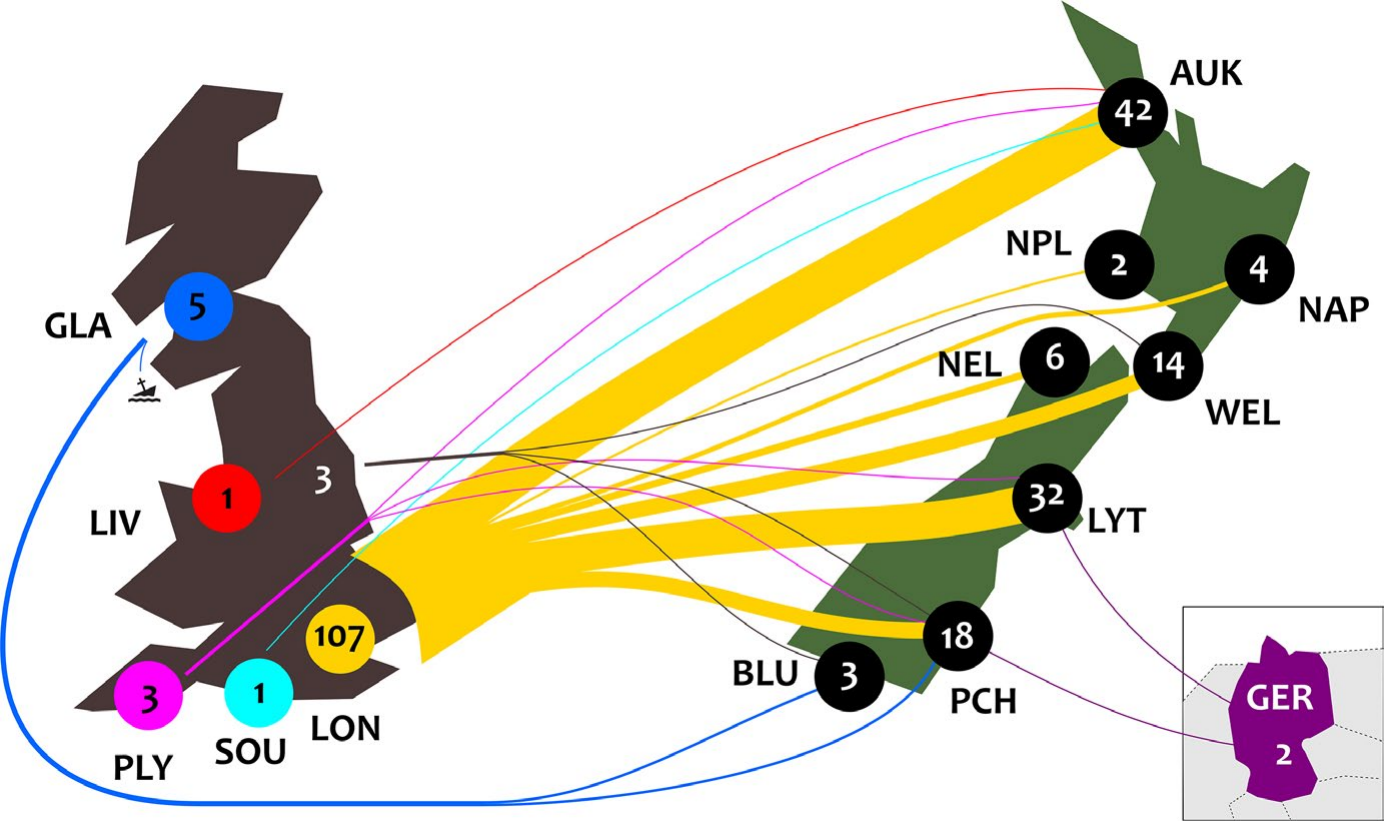
Between 1850 and 1884, passerines were shipped at least 122 times from Europe, mostly from port cities in England, to New Zealand. One ship was wrecked not far from the start. The thickness of the lines corresponds to the number of ships that travelled between the ports which are connected by them, and the colors serve only to distinguish the lines from different ports from each other. The brown and the purple correspond to England and Germany, respectively, and the port is unknown. The destinations are abbreviated as follows (NZ regions are in brackets): AUK—Auckland (Auckland), BLU—Bluff (Southland), GLA—Glasgow, LIV—Liverpool, LON—London, LYT—Lyttelton (Canterbury), NAP—Napier (Hawke's Bay), NEL—Nelson (Nelson), NPL—New Plymouth (Taranaki), PCH—Port Chalmers (Otago), PLY—Plymouth, SOU—Southampton, WEL—Wellington (Wellington)

Most of the 122 shipments we found departed from London (*n* = 107), with the remainder from other UK ports, and two from Germany, likely from Hamburg (Figure 1). Of these shipments, 36.9% (45 ships) went to Auckland and 25.4% (31 ships) to Canterbury, with the rest distributed across other New Zealand ports (Figure 1). The number of species transported was highest to Canterbury, Otago, and Auckland, with smaller numbers imported at other locations in New Zealand (Table 1). The most diverse shipment (on the British Empire in 1864) contained 17 species of passerines, but the median number was three species per ship. Some of the species (e.g., skylarks, blackbirds, starlings *Sturnus vulgaris,* or thrushes) travelled on dozens of ships, while some travelled just once or twice (e.g., cirl buntings *Emberiza cirlus* and ravens *Corvus corax*); the median number of shipments per species was 6.

**Table 1:** Summary statistics for different NZ ports

Port	Region	Number of ships	Number of species	Median survival
Auckland	Auckland	42	23	0.18 (18)
Bluff	Southland	3	7	
Lyttelton	Canterbury	32	29	0.29 (7)
Napier	Hawke's Bay	4	15	
Nelson	Nelson	6	12	0.33 (5)
Port Chalmers	Otago	18	28	0.60 (3)
New Plymouth	Taranaki	2	8	
Wellington	Wellington	14	16	0.00 (3)

Note

Median survival was counted only for a subset of shipments (*n* = 37) and for taxa as specified in methods (number of shipments shown in brackets).

### 3.2 Survival during transport

**Figure 2:**
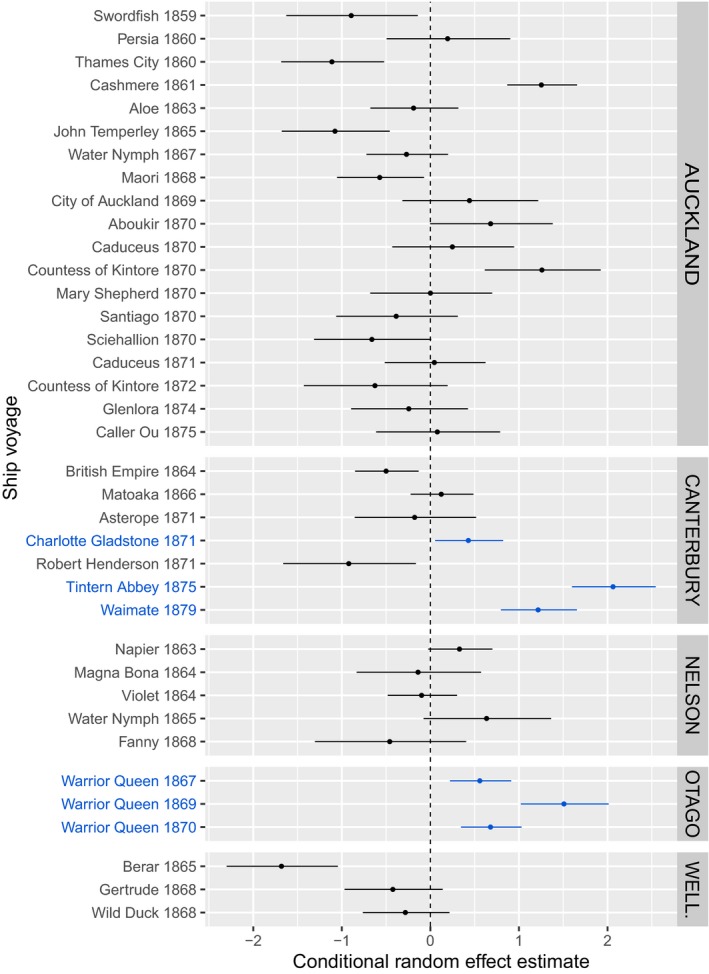
Conditional estimates of probability of survival (log odds scale) for ship voyages to five different regions in New Zealand; error bars are standard errors. Positive and negative values with standard error not overlapping the zero line indicate significantly higher survival or mortality, respectively. Zero value indicates no deviation from the average survival conditional on the other modeled effects. Shipments highlighted in blue were organized and accompanied by Richard Bills or his son Henry

**Figure 3:**
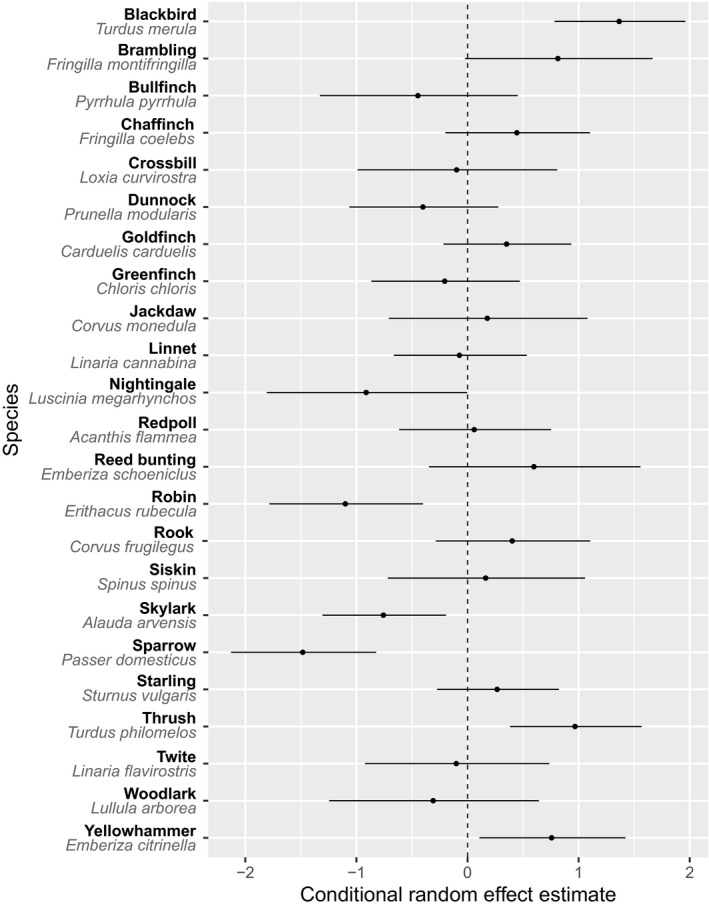
Conditional estimates of probability of survival (log odds scale) for bird species; error bars are standard errors. Positive and negative values with standard error not overlapping the zero line indicate significantly higher survival or mortality, respectively. Zero value indicates no deviation from the average survival conditional on the other modeled effects

The median survival rate across species and voyages was 0.28 (95% CI = 0.18, 0.41), ranging from zero survival (on the Berar in 1865) to 78% of loaded birds completing the voyage (on the Tintern Abbey in 1875). There was no evidence for systematic differences in survival associated with either the duration or the season of the voyage, but we found an overall trend for an increase in average survival over time (Table 2). There was approximately a 12% increase in the average odds of survival in any year relative to the previous year (95% CI = 2%–24%). Most of the variation in survival was among voyages (*SD* = 1.03, Figure 2) and among species (*SD* = 0.54, Figure 3). We found no relationships between survival and any of the species traits we examined (Table 3).

**Table 2:** Analysis of deviance table for nested model comparisons using likelihood ratio tests

Model comparison	Number of parameters	Log‐likelihood	Deviance	*df*	*p*‐Value
Duration + year + season	9	−448.64	4.94	6	.55
ns(Duration, *df* = 3) + ns(year, *df* = 5) + season	15	−446.17			
Duration	5	−452.29	7.28	4	.12
Duration + year + season	9	−448.64			
Year	5	−450.00	2.72	4	.61
Duration + year + season	9	−448.64			
Season	7	−450.09	2.89	2	.24
Duration + year + season	9	−448.64			
Intercept only	4	−452.43	0.29	1	.59
Duration	5	−452.29			
Intercept only	4	−452.43	4.86	1	.03
Year	5	−450.00			
Intercept only	4	−452.43	4.69	3	.20
Season	7	−450.09			

Note

All models, that is, generalized linear mixed models (with beta‐binomial logit function) with proportion of surviving bird individuals out of the total loaded as a response variable, contain an intercept term; “ns” indicates natural spline. Difference in model deviance is assumed to be distributed as chi‐squared with specified degrees of freedom (*df*).

**Table 3:** Analysis of deviance tables for nested model comparisons for species trait variables, one continuous—body mass, three binaries—sexual dimorphism, sedentary, and short‐distance migrant (the latter together describe migratory behavior—two zeroes equal to long‐distance migration), and one categorial—the diet (possible values insectivorous, omnivorous, plant/seed eater)

Model comparison	Number of parameters	Log‐likelihood	Deviance	*df*	*p*‐Value
Year	5	−450.00	1.04	1	.31
Year + body mass	6	−449.49			
Year	5	−450.00	1.84	1	.17
Year + sexual dimorphism	6	−449.08			
Year	5	−450.00	0.20	1	.65
Year + sedentary	6	−449.90			
Year	5	−450.00	2.96	1	.09
Year + short‐distance migrant	6	−448.53			
Year	5	−450.00	1.72	2	.42
Year + diet	7	−449.15			

Note

All models, that is, generalized linear mixed models (with beta‐binomial logit function) with proportion of surviving bird individuals out of the total loaded as a response variable, contain an intercept term. Difference in model deviance is assumed to be distributed as chi‐squared with specified degrees of freedom (*df*).

## 4 DISCUSSION

We built a unique dataset of 122 ships carrying passerines from Europe (almost exclusively from Great Britain) to New Zealand. The dataset is certainly not complete—some species were clearly shipped without leaving a transport record, for example six ortolan buntings *Emberiza hortulana* brought in 1884 to Wellington from the South of France (Wellington & Wairarapa district Acclimatisation Society, 1885). Nevertheless, it is the most robust dataset ever built concerning the historical shipment of alien birds to New Zealand, and, to our best knowledge, anywhere. For one‐third of these shipments, we were able to analyze the factors affecting bird survival during transport.

We did not find predictors of variation in the likelihood of passerine birds surviving the voyage to New Zealand, with the exception of an overall trend toward higher survival rates over time. This trend was probably driven by early failures, subsequently leading to improved policies, and by the fact that initial (and not very successful) private endeavors (Canterbury Acclimatisation Society, 1866) were, in the late 1860s and in 1870s, replaced by large shipments organized by the Acclimatisation Societies, with very low mortality (e.g., Warrior Queen in 1868, 1870 and 1871 to Otago and Charlotte Gladstone in 1872, Tintern Abbey in 1875, and Waimate in 1880 to Canterbury). For all these last mentioned shipments, birds were collected and later accompanied on board by the very experienced bird fancier from Brighton (South England), Richard Bills, whose services in this regard were much prized by the Societies (e.g., Carrick, 1870; Farr, 1870; Murison, 1871), or by his son Henry (who accompanied the most successful shipment on the Tintern Abbey). This implies that it was the quality of care received by the birds, both prior to and during shipment, that most affected success at this stage of the invasion process. Indeed, when we compared the success of shipments accompanied by either Richard or Henry Bills with other shipments departing in the same period (i.e., in the years 1867, 1869, 1870, 1871, 1875, 1879), their presence significantly improved bird survival rates (likelihood ratio test contrasting models containing the binary classification of voyages where Bills cared for the birds vs. not: deviance = 7.0, *df* = 1, *p* = .008, Table S4).

No other features of the shipments explained variation in the bird survival. Although it was suggested several times during the 19th century (e.g., Brodie, 1859; Hawke's Bay Herald, 1865) that it is crucial to collect birds outside their breeding season for transportation, our analyses did not provide any evidence that the timing of departure mattered. Neither was the length of the voyage important, at least within the range of variation encompassed by these sailings (90–139 days).

Survival rate on these voyages varied among taxa: Some species, such as song thrushes, blackbirds, and bramblings *Fringilla montifringilla*, appeared to cope quite well with the long transport, while other species, such as robins or house sparrows *Passer domesticus*, had exceptionally high mortality rates. For the sparrows, this matches their chances of surviving voyages to Australia (Andrew & Griffith, 2016). Nevertheless, we did not find any evidence that interspecific differences in survival were associated with traits of the species, at least those analyzed here. The lack of an effect of body mass was surprising, as larger (e.g., blackbirds, thrushes, and rooks) and smaller birds were sometimes treated differently. For example, these large birds were put individually into cages for the voyage (Press, 1867a), while rooks, thrushes, and blackbirds sometimes had their wing feathers cut (Auckland Acclimatisation Society, 1871; Press, 1867c), and upon arrival, they had to be kept in aviaries (Press, 1875b) to allow these feathers to regrow before being released. The nonsignificant effects of other traits may be due to the low sample size for some of them—for example, there was only one strictly migratory species (nightingale *Luscinia megarhynchos*) in our data, constraining the amount of variation this trait could explain. It is also possible that we did not analyze the key traits distinguishing species with high and low survival rates, although it is difficult to predict what these might be.

This study highlights that by not considering the transport stage of the invasion pathway, we may lose information relevant to the invasion process. For example, a species might appear not to have been selected for introduction, but it could in fact have been entrained in the transport step, only to fail to survive the voyage (which appears to be the case for blackcaps *Sylvia atricapilla* departing in 1867 on Warrior Queen). Alternatively, a species could survive the trip, but not be released. This was the case for several species in a shipment on the Waimate, which arrived in 1880 with a different set of species than had been requested by the Acclimatisation Society. Although it was the first shipment of cirl buntings to Lyttelton, the Society no longer wanted this species, nor its close relative, the yellowhammer (Pipek et al., 2015). A group of bramblings on this ship was also refused, and this decision may have been responsible for them not having established—as many as 107 arrived on the Waimate (probably many more than in all other seven shipments of the species combined), which would typically be enough to allow a species to establish (Cassey, Delean, Lockwood, Sadowski, & Blackburn, 2018; Duncan, Blackburn, Rossinelli, & Bacher, 2014). In the case of crossbills *Loxia curvirostra*, the refusal may be even more significant, as this was the only shipment of this species to New Zealand, though the number of individuals (*n* = 12; Lyttelton Times, 1880) might not have been sufficient for establishment.

Knowledge of the transport process may also improve our understanding of subsequent invasion stages in other ways. For example, the surviving individuals of a species, which experienced high mortality during transportation, may not have arrived in the best condition (Nelson Examiner & New Zealand Chronicle, 1864; Press, 1873), and therefore, the chances of their subsequent establishment might have been hampered. A particularly low survival rate (Press, 1875a) may have contributed to the failure of robins, which were shipped quite frequently (29 shipments), but never established. That said, along with nightingales, robins continued to be shipped long after acclimatization ceased to be popular (robins in 1899, nightingales in 1928), but to no avail. In contrast, sparrows were also evidently not very good at surviving on boats, but despite that, became a widespread and prolific invader. This showcases the fact that different selection pressures might be in play in different stages of an invasion, and that a lack of success in one stage (e.g., transport, introduction) does not imply that a species will also fail at other stages (e.g., establishment, spread), if given the opportunity (Blackburn et al., 2011).

For robins, a further reason suggested for their failure to establish was that only males were shipped. However, robins are not easy to sex, and this information was already considered dubious by Thomson (1922) who conveyed it from its source, Mr. A. Binnie. Nevertheless, sex ratio was sometimes significantly skewed toward males in other species in some shipments (e.g., William Davie and Matoaka in 1867, Maori in 1869 and Warrior Queen in 1868 and 1870; Otago Daily Times, 1867; Press, 1867b; New Zealand Herald, 1869; Evening Star, 1870), and there is also some evidence that females tended to survive transportation worse (as noted in the Otago Daily Times, 1867). This could result in a lower effective population size at introduction for some species, affecting their probability of establishment. Indeed, the Otago Acclimatisation Society in 1867 even ordered more females than males in shipments, following their previous bad experiences (Otago Daily Times, 1867). It is possible that interspecific and intership variation in survival rates could be a consequence of variation in sex ratio between shipments, but unfortunately there is insufficient information on the sex ratios of birds in shipments to test this hypothesis.

In conclusion, we have shown that there was substantial variation between shipments and species in the survival rate of birds transported from Europe to New Zealand in the second half of the 19th century, but besides increased survival rate with time, we could identify no predictors of this variation. However, our analysis was conducted only on a subset of all shipments and for only some of the transported species. It might be advantageous to extend our analysis on a larger dataset including records of shipments to other countries with the same species, from the same source, for example, Australia and the United States, as these countries also differed in the establishment success of the same bird species (Moulton & Cropper, 2014). We limited our analysis to passerines to constrain the scope of the study, both in terms of time period and the number of source articles to review, to manageable levels (c.f. Duncan, 1997; Green, 1997), but including species from a wider range of bird taxa may capture more variation in specific traits that might help explain some of the variation in success. Nevertheless, even on our reduced sample of species and shipments, we have demonstrated variation in survival that may be pertinent to our understanding of the invasion process.

## CONFLICT OF INTEREST

None declared.

## AUTHOR CONTRIBUTIONS

PPi conceived the idea and collected most of the data. PPi, PPy, TMB, and PC developed the design and were involved both in data analysis and drafting the manuscript. SD analyzed the data and contributed to drafting and revising of the MS. ÇHŞ provided important source of data about bird traits and revised the manuscript critically. All the authors have commented on the final version of the manuscript and approved it.

## Data Availability

The data on shipments used in the analysis are stored in Table S1. The data on bird traits coming partly from the dataset by Storchová and Hořák (2018), partly provided by ÇHŞ, are stored in Table S2.

## References

[ece36143-bib-0001] Andrew, S. C. , & Griffith, S. C. (2016). Inaccuracies in the history of a well‐known introduction: A case study of the Australian House Sparrow (*Passer domesticus*). Avian Research, 7, 9 10.1186/s40657-016-0044-3

[ece36143-bib-0002] Auckland Acclimatisation Society (1871). Report of the Auckland Acclimatisation Society for the year ending February 28, 1871. Auckland, New Zealand: New Zealand Herald.

[ece36143-bib-0003] Bellard, C. , Genovesi, P. , & Jeschke, J. M. (2016). Global patterns in threats to vertebrates by biological invasions. Proceedings of the Royal Society B: Biological Sciences, 283(1823), 20152454 10.1098/rspb.2015.2454 PMC4795027 26817767

[ece36143-bib-0004] Blackburn, T. M. , Pyšek, P. , Bacher, S. , Carlton, J. T. , Duncan, R. P. , Jarošík, V. , … Richardson, D. M. (2011). A proposed unified framework for biological invasions. Trends in Ecology & Evolution, 26(7), 333–339. 10.1016/j.tree.2011.03.023 21601306

[ece36143-bib-0005] Brodie, W. (1859). Importation of English birds and forest trees into Australia. Bell's life in Tasmania, Hobart Town, 11 October 1859. Retrieved from https://trove.nla.gov.au/newspaper/article/232483608

[ece36143-bib-0006] Canterbury Acclimatisation Society (1866). The Second Annual Report of the Canterbury Acclimatisation Society. Christchurch.

[ece36143-bib-0007] Carrick, A. (Otago Acclimatisation Society ) (1870). Letter to S. Farr (Canterbury Acclimatisation Society) from 6 June. In Letter Book, 1867–1878, Otago Acclimatisation Society, 93‐023/54. Dunedin, New Zealand: Hocken Collections.

[ece36143-bib-0008] Cassey, P. , Blackburn, T. M. , Russell, G. J. , Jones, K. E. , & Lockwood, J. L. (2004). Influences on the transport and establishment of exotic bird species: An analysis of the parrots (Psittaciformes) of the world. Global Change Biology, 10(4), 417–426. 10.1111/j.1529-8817.2003.00748.x

[ece36143-bib-0009] Cassey, P. , Delean, S. , Lockwood, J. L. , Sadowski, J. S. , & Blackburn, T. M. (2018). Dissecting the null model for biological invasions: A meta‐analysis of the propagule pressure effect. PLOS Biology, 16(4), e2005987 10.1371/journal.pbio.2005987 29684017 PMC5933808

[ece36143-bib-0010] Clavero, M. , & Garcia‐Berthou, E. (2005). Invasive species are a leading cause of animal extinctions. Trends in Ecology & Evolution, 20(3), 110–110. 10.1016/j.tree.2005.01.003 16701353

[ece36143-bib-0011] di Castri, F. (1989). History of biological invasions with special emphasis on the Old World In Drake J. A., Mooney H. A., di Castri F., Groves R. H., Kruger F., Rejmánek M., & Williamson M. (Eds.), Biological invasions: A global perspective (pp. 1–30). Chichester, UK: John Wiley and Sons.

[ece36143-bib-0012] Drummond, J. (1906). On introduced birds. Transactions of the New Zealand Institute, 39, 503–508.

[ece36143-bib-0013] Duncan, R. P. (1997). The role of competition and introduction effort in the success of Passeriform birds introduced to New Zealand. The American Naturalist, 149(5), 903–915. 10.1086/286029 18811254

[ece36143-bib-0014] Duncan, R. P. , Blackburn, T. M. , & Cassey, P. (2006). Factors affecting the release, establishment and spread of introduced birds in New Zealand In Allen R. B., & Lee W. G. (Eds.), Biological invasions in New Zealand (pp. 137–154). Berlin and Heidelberg, Germany: Springer, Berlin Heidelberg.

[ece36143-bib-0015] Duncan, R. P. , Blackburn, T. M. , Rossinelli, S. , & Bacher, S. (2014). Quantifying invasion risk: The relationship between establishment probability and founding population size. Methods in Ecology and Evolution, 5(11), 1255–1263.

[ece36143-bib-0016] Duncan, R. P. , Blackburn, T. M. , & Sol, D. (2003). The ecology of bird introductions. Annual Review of Ecology, Evolution, and Systematics, 34, 71–98. 10.1146/annurev.ecolsys.34.011802.132353

[ece36143-bib-0017] Dyer, E. E. , Redding, D. W. , & Blackburn, T. M. (2017). The global avian invasions atlas, a database of alien bird distributions worldwide. Scientific Data, 4, 170041 10.1038/sdata.2017.41 28350387 PMC5369319

[ece36143-bib-0018] Evening Star (1870). Sales by auction. Evening Star, VIII(2112), 3, 11 February. Retrieved from https://paperspast.natlib.govt.nz/newspapers/ESD18700211.2.17.1

[ece36143-bib-0019] Farr, S. (1870). Untitled letter to Richard Bills from 29 April. In Canterbury Acclimatisation Society, Letter Books (1869–1884), ANZC Archives (ARCHIVE 5 1). Christchurch, New Zealand: Tūranga (Central Library).

[ece36143-bib-0020] Farr, S. (1872). Letter to A. O. Ottywell from 15 February. In Canterbury Acclimatisation Society, Letter Books (1869–1884), ANZC Archives (ARCHIVE 5 1). Christchurch, New Zealand: Tūranga (Central Library).

[ece36143-bib-0021] Green, R. (1997). The influence of numbers released on the outcome of attempts to introduce exotic bird species to New Zealand. Journal of Animal Ecology, 66(1), 25–35. 10.2307/5961

[ece36143-bib-0022] Hawke's Bay Herald (1865). Importation of birds. Hawke's Bay Herald, 8(682), 3, 12 September 1865. Retrieved from https://paperspast.natlib.govt.nz/newspapers/HBH18650912.2.13

[ece36143-bib-0023] Kiat, Y. , Vortman, Y. , & Sapir, N. (2019). Feather moult and bird appearance are correlated with global warming over the last 200 years. Nature Communications, 10, 2540 10.1038/s41467-019-10452-1 PMC6557852 31182713

[ece36143-bib-0024] Lever, C. (1992). They dined on eland: The story of the Acclimatization Societies (1st. ed.). London, UK: Quiller Press.

[ece36143-bib-0025] Lockwood, J. L. , & McKinney, M. L. (2001). Biotic homogenization. New York, NY: Springer Science & Business Media.

[ece36143-bib-0026] Long, J. L. (1981). Introduced birds of the world. Newton Abbot, UK: David & Charles.

[ece36143-bib-0027] Lonsdale, W. M. (1999). Global patterns of plant invasions and the concept of invasibility. Ecology, 80(5), 1522 10.1890/0012-9658(1999)080[1522:GPOPIA]2.0.CO;2

[ece36143-bib-0028] Lyttelton Times (1880). Acclimatisation society. Lyttelton times, LIII(5936), 5, 5 March. Retrieved from https://paperspast.natlib.govt.nz/newspapers/LT18800305.2.27

[ece36143-bib-0029] McDowall, R. M. (1994). Gamekeepers for the nation: The story of New Zealand's acclimatisation societies, 1861–1990. Christchurch, UK: Canterbury University Press.

[ece36143-bib-0030] Morrison, C. A. , Baillie, S. R. , Clark, J. A. , Johnston, A. , Leech, D. I. , & Robinson, R. A. (2015). Flexibility in the timing of post‐breeding moult in passerines in the UK. Ibis, 157(2), 340–350. 10.1111/ibi.12234

[ece36143-bib-0031] Moulton, M. P. , & Cropper, W. P. (2014). A comparison of success rates of introduced passeriform birds in New Zealand, Australia and the United States. PeerJ, 2, e509 10.7717/peerj.509 25165625 PMC4137686

[ece36143-bib-0032] Murison, W. (Nelson Acclimatisation Society ) (1871). Letter to S. Farr (Canterbury Acclimatisation Society) from 26 April. In Letter Books (1869–1884), Canterbury Acclimatisation Society, ANZC Archives (ARCHIVE 5 1). Christchurch, New Zealand:Tūranga (Central Library).

[ece36143-bib-0033] Nelson Examiner and New Zealand Chronicle (1864). Acclimatisation society. Nelson Examiner and New Zealand Chronicle, XXIII(110), 2, 12 September. Retrieved from https://paperspast.natlib.govt.nz/newspapers/NENZC18640912.2.14

[ece36143-bib-0034] New Zealand Herald (1869). Acclimatisation. Importation of English birds. New Zealand Herald, VI(1637), 3, 19 February. Retrieved from https://paperspast.natlib.govt.nz/newspapers/NZH18690219.2.26

[ece36143-bib-0035] Otago Daily Times (1867). Social and domestic. Otago Daily times, (1838), 5, 20 November. Retrieved from https://paperspast.natlib.govt.nz/newspapers/ODT18671120.2.24

[ece36143-bib-0036] Peoples, B. K. , & Goforth, R. R. (2017). The indirect role of species‐level factors in biological invasions. Global Ecology and Biogeography, 26(5), 524–532. 10.1111/geb.12567

[ece36143-bib-0037] Peters, R. (1983). The ecological implications of body size. Cambridge, UK: Cambridge University Press.

[ece36143-bib-0038] Pipek, P. , Blackburn, T. M. , & Pyšek, P. (2019). The ins and outs of acclimatisation: Imports versus translocations of skylarks and starlings in 19th century New Zealand. Biological Invasions, 21(4), 1395–1413. 10.1007/s10530-018-1905-y

[ece36143-bib-0039] Pipek, P. , Pyšek, P. , & Blackburn, T. M. (2015). How the Yellowhammer became a Kiwi: The history of an alien bird invasion revealed. NeoBiota, 24, 1–31. 10.3897/neobiota.24.8611

[ece36143-bib-0040] Press (1867a). Acclimatisation. Press, XI(1310), 2, 18 January. Retrieved from https://paperspast.natlib.govt.nz/newspapers/CHP18670118.2.12

[ece36143-bib-0041] Press (1867b). Acclimatisations society. Press, XI(1371), 2, 30 March. Retrieved from https://paperspast.natlib.govt.nz/newspapers/CHP18670330.2.17

[ece36143-bib-0042] Press (1867c). Treatment of small birds on long voyages. Press, XI(1371), 3, 30 March. Retrieved from https://paperspast.natlib.govt.nz/newspapers/CHP18670330.2.21

[ece36143-bib-0043] Press (1873). Acclimatisation society. Press, XXI(2373), 3, 13 March. Retrieved from https://paperspast.natlib.govt.nz/newspapers/CHP18730313.2.19

[ece36143-bib-0044] Press (1875a). Departure from England of the Tintern Abbey. Press, XXIII(2996), 2, 30 March. Retrieved from https://paperspast.natlib.govt.nz/newspapers/CHP18750330.2.7

[ece36143-bib-0045] Press (1875b). Acclimatisation society. Press, XXIII(3054), 3, 5 June. Retrieved from https://paperspast.natlib.govt.nz/newspapers/CHP18750605.2.12

[ece36143-bib-0046] Promislow, D. E. L. , Montgomerie, R. , & Martin, T. E. (1992). Mortality costs of sexual dimorphism in birds. Proceedings of the Royal Society of London. Series B: Biological Sciences, 250(1328), 143–150.

[ece36143-bib-0047] R Core Team (2018). R: A language and environment for statistical computing. Vienna, Austria: R Foundation for Statistical Computing Retrieved from https://www.r-project.org

[ece36143-bib-0048] Seebens, H. , Blackburn, T. M. , Dyer, E. E. , Genovesi, P. , Hulme, P. E. , Jeschke, J. M. , … Essl, F. (2017). No saturation in the accumulation of alien species worldwide. Nature Communications, 8, 14435 10.1038/ncomms14435 PMC5316856 28198420

[ece36143-bib-0049] Şekercioğlu, Ç. H. , Mendenhall, C. D. , Brenes, F. O. , Horns, J. J. , Ehrlich, P. R. , & Daily, G. C. (2019). Long‐term declines in bird populations in tropical agricultural countryside. Proceedings of the National Academy of Sciences of the United States of America, 116(20), 9903–9912. 10.1073/pnas.1802732116 31036662 PMC6525491

[ece36143-bib-0050] Skaug, H. , Fournier, D. , Bolker, B. , Magnusson, A. , & Nielsen, A. (2016). Generalized linear mixed models using ‘AD Model Builder’. R package version 0.8.4.

[ece36143-bib-0051] Sorci, G. , Moller, A. P. , & Clobert, J. (1998). Plumage dichromatism of birds predicts introduction success in New Zealand. Journal of Animal Ecology, 67(2), 263–269. 10.1046/j.1365-2656.1998.00199.x

[ece36143-bib-0052] Star, P. (2014). Human agency and exotic birds in New Zealand. Environment and History, 20(2), 275–299. 10.3197/096734014X13941952681070

[ece36143-bib-0053] Storchová, L. , & Hořák, D. (2018). Life‐history characteristics of European birds. Global Ecology and Biogeography, 27(4), 400–406. 10.1111/geb.12709

[ece36143-bib-0054] Su, S. , Cassey, P. , & Blackburn, T. M. (2014). Patterns of non‐randomness in the composition and characteristics of the Taiwanese bird trade. Biological Invasions, 16(12), 2563–2575. 10.1007/s10530-014-0686-1

[ece36143-bib-0055] Thomson, G. (1922). The naturalisation of plants and animals in New Zealand. London, UK: Cambridge University Press.

[ece36143-bib-0056] Wellington and Wairarapa District Acclimatisation Society (1885). First annual report of Wellington and Wairarapa district Acclimatisation Society. Wellington, New Zealand: James Hughes.

