# Supplementary material for: Lasting the distance: The survival of alien birds shipped to New Zealand in the 19th century
Source: Ecol Evol. 2020 Mar 7;10(9):3944–53. doi: 10.1002/ece3.6143 (PMC7244811; doi:10.1002/ece3.6143)
Supplement: Supplementary file 1 [file ECE3-10-3944-s001.docx]

**Supplementary table 1:** Shipments and associated taxa that were used in our analysis of survival, 37 shipments, with 23 species on board were used.

| **Name of the Ship** | **Year of departure** | **Length of the voyage (days)** | **Season of departure** | **Species** | **Loaded** | **Survived** | **Mortality cause** | **Reference** |
| --- | --- | --- | --- | --- | --- | --- | --- | --- |
| Swordfish | 1859 | 132 | 61 | sparrow | 360 | 0 | sparrows were shipped in only five cages | Colonist 1859 |
|  |  |  |  |  |  |  |  |  |
| Persia | 1860 | 139 | 122 | linnet | 48 | 15 |  | New Zealander 1860a |
|  |  |  |  |  |  |  |  |  |
| Thames City | 1860 | 102 | 208 | goldfinch | 6 | 0 |  | New Zealander 1860b |
|  |  |  |  | linnet | 6 | 0 |  |  |
|  |  |  |  | skylark | 60 | 3 |  |  |
|  |  |  |  | starling | 36 | 0 |  |  |
|  |  |  |  |  |  |  |  |  |
| Cashmere | 1861 | 118 | 344 | linnet | 9 | 8 | limited space | Daily Southern Cross 1862 |
|  |  |  |  | robin | 6 | 6 |  |  |
|  |  |  |  | skylark | 12 | 10 |  |  |
|  |  |  |  | thrush | 13 | 11 |  |  |
|  |  |  |  | sparrow | 16 | 7 |  |  |
|  |  |  |  | starling | 12 | 9 |  |  |
|  |  |  |  | blackbird | 12 | 10 |  |  |
|  |  |  |  | bullfinch | 8 | 3 |  |  |
|  |  |  |  | goldfinch | 8 | 5 |  |  |
|  |  |  |  | chaffinch | 16 | 6 |  |  |
|  |  |  |  |  |  |  |  |  |
| Aloe | 1863 | 122 | 36 | skylark | 11 | 1 | large number died before leaving Gravesend | King 1863; Morrin 1863 |
|  |  |  |  | sparrow | 27 | 0 |  |  |
|  |  |  |  | redpoll | 2 | 0 |  |  |
|  |  |  |  | starling | 9 | 2 |  |  |
|  |  |  |  | dunnock | 8 | 4 |  |  |
|  |  |  |  |  |  |  |  |  |
| Napier | 1863 | 94 | 248 | linnet | 8 | 2 | all sparrows died early on the passage | Colonist 1863 |
|  |  |  |  | robin | 9 | 1 |  |  |
|  |  |  |  | skylark | 51 | 23 |  |  |
|  |  |  |  | thrush | 5 | 3 |  |  |
|  |  |  |  | sparrow | 48 | 0 |  |  |
|  |  |  |  | starling | 18 | 9 |  |  |
|  |  |  |  | greenfinch | 9 | 4 |  |  |
|  |  |  |  | blackbird | 34 | 21 |  |  |
|  |  |  |  | goldfinch | 9 | 5 |  |  |
|  |  |  |  |  |  |  |  |  |
| British Empire | 1864 | 98 | 152 | rook | 18 | 0 | limited space, the boat was not prepared for the birds | Lyttelton Times 1864 |
|  |  |  |  | blackbird | 18 | 1 |  |  |
|  |  |  |  | skylark | 66 | 2 |  |  |
|  |  |  |  | brambling | 3 | 3 |  |  |
|  |  |  |  | bullfinch | 2 | 0 |  |  |
|  |  |  |  | starling | 8 | 1 |  |  |
|  |  |  |  | wood lark | 6 | 0 |  |  |
|  |  |  |  | goldfinch | 26 | 2 |  |  |
|  |  |  |  | yellowhammer | 12 | 2 |  |  |
|  |  |  |  | greenfinch | 11 | 3 |  |  |
|  |  |  |  | chaffinch | 4 | 1 |  |  |
|  |  |  |  | sparrow | 72 | 0 |  |  |
|  |  |  |  | jackdaw | 1 | 0 |  |  |
|  |  |  |  | linnet | 47 | 8 |  |  |
|  |  |  |  | robin | 7 | 3 |  |  |
|  |  |  |  |  |  |  |  |  |
| Violet | 1864 | 111 | 76 | linnet | 48 | 8 |  | New Zealand Herald 1864; Otago Witness 1864 |
|  |  |  |  | skylark | 24 | 0 |  |  |
|  |  |  |  | thrush | 6 | 3 |  |  |
|  |  |  |  | sparrow | 72 | 2 |  |  |
|  |  |  |  | starling | 42 | 12 |  |  |
|  |  |  |  | blackbird | 18 | 7 |  |  |
|  |  |  |  | greenfinch | 48 | 16 |  |  |
|  |  |  |  |  |  |  |  |  |
| Berar | 1865 | 93 | 36 | chaffinch | 20 | 0 |  | Wellington Independent 1865 |
|  |  |  |  | linnet | 20 | 0 |  |  |
|  |  |  |  | thrush | 6 | 0 |  |  |
|  |  |  |  | greenfinch | 20 | 0 |  |  |
|  |  |  |  | skylark | 6 | 0 |  |  |
|  |  |  |  |  |  |  |  |  |
| John Temperley | 1864 | 114 | 128 | blackbird | 30 | 4 | birds were nesting at the time of shipment | Hawke's Bay Herald 1865 |
|  |  |  |  | thrush | 30 | 0 |  |  |
|  |  |  |  |  |  |  |  |  |
| Magna Bona | 1864 | 105 | 341 | sparrow | 48 | 1 |  | Nelson Examiner and New Zealand Chronicle 1865. |
|  |  |  |  |  |  |  |  |  |
| Water nymph | 1865 | 104 | 241 | rook | 8 | 6 |  | Taranaki Herald 1866 |
|  |  |  |  |  |  |  |  |  |
| Matoaka | 1866 | 95 | 280 | blackbird | 48 | 46 | small birds - overcrowded (the blackbirds and thrushes were put separately), lack of experienced attendant | Press 1866; Lyttelton Times 1867 |
|  |  |  |  | greenfinch | 48 | 0 |  |  |
|  |  |  |  | robin | 12 | 0 |  |  |
|  |  |  |  | dunnock | 10 | 1 |  |  |
|  |  |  |  | skylark | 96 | 13 |  |  |
|  |  |  |  | chaffinch | 36 | 11 |  |  |
|  |  |  |  | thrush | 48 | 36 |  |  |
|  |  |  |  | linnet | 72 | 21 |  |  |
|  |  |  |  | starling | 50 | 20 |  |  |
|  |  |  |  |  |  |  |  |  |
| Water nymph | 1867 | 93 | 234 | starling | 200 | 11 |  | Daily Southern Cross 1867a,b |
|  | 1867 |  |  | greenfinch | 50 | 5 |  |  |
|  | 1867 |  |  | skylark | 150 | 3 |  |  |
|  | 1867 |  |  | sparrow | 200 | 41 |  |  |
|  |  |  |  |  |  |  |  |  |
| Gertrude | 1868 | 107 | 50 | skylark | 120 | 1 |  | Nelson Examiner and New Zealand Chronicle 1868 |
|  |  |  |  | starling | 40 | 6 |  |  |
|  |  |  |  |  |  |  |  |  |
| Warrior Queen | 1867 | 99 | 294 | skylark | 214 | 37 | water in the deck | Oamaru Times 1867; Wellington Independent 1868; Otago Acclimatisation Society (unpublished) |
|  |  |  |  | blackbird | 76 | 63 |  |  |
|  |  |  |  | goldfinch | 116 | 40 |  |  |
|  |  |  |  | starling | 228 | 107 |  |  |
|  |  |  |  | greenfinch | 15 | 8 |  |  |
|  |  |  |  | yellowhammer | 12 | 8 |  |  |
|  |  |  |  | chaffinch | 78 | 55 |  |  |
|  |  |  |  | thrush | 103 | 94 |  |  |
|  |  |  |  | robin | 37 | 0 |  |  |
|  |  |  |  |  |  |  |  |  |
| Fanny | 1868 | 126 | 349 | rook | 2 | 0 |  | Nelson Evening Mail 1869 |
|  |  |  |  |  |  |  |  |  |
| Maori | 1868 | 122 | 292 | skylark | 182 | 1 |  | Daily Southern Cross 1869; New Zealand Herald 1869b |
|  |  |  |  | starling | 400 | 41 |  |  |
|  |  |  |  | chaffinch | 218 | 16 |  |  |
|  |  |  |  |  |  |  |  |  |
| Wild Duck | 1868 | 96 | 275 | skylark | 84 | 1 |  | Colonist 1868; Hawke's Bay Herald 1869; New Zealand Herald 1869a |
|  |  |  |  | sparrow | 84 | 9 |  |  |
|  |  |  |  | starling | 100 | 65 |  |  |
|  |  |  |  | dunnock | 50 | 0 |  |  |
|  |  |  |  |  |  |  |  |  |
|  |  |  |  |  |  |  |  |  |
| City of Auckland | 1869 | 102 | 292 | rook | 3 | 2 |  | New Zealand Herald 1869c[; New Zealand Herald 1870a](https://paperspast.natlib.govt.nz/newspapers/NZH18700131.2.25) |
|  |  |  |  |  |  |  |  |  |
| Warrior Queen | 1869 | 95 | 305 | starling | 95 | 94 |  | Oamaru Times 1870; Carrick 1870 |
|  |  |  |  |  |  |  |  |  |
| Aboukir | 1870 | 97 | 152 | rook | 24 | 18 | fed by preserved meat | Daily Southern Cross 1870b |
|  |  |  |  |  |  |  |  |  |
| Countess of Kintore | 1870 | 97 | 180 | rook | 50 | 48 |  | Daily Southern Cross 1870c |
|  |  |  |  |  |  |  |  |  |
| Mary Shepherd | 1870 | 117 | 32 | yellowhammer | 120 | 40 |  | Daily Southern Cross 1870a; New Zealand Herald 1870b |
|  |  |  |  |  |  |  |  |  |
| Sciehallion | 1870 | 98 | 57 | yellowhammer | 128 | 5 |  | Auckland Star 1870; New Zealand Herald 1870c |
|  |  |  |  |  |  |  |  |  |
|  |  |  |  | goldfinch | 60 | 50 |  |  |
|  |  |  |  | skylark | 60 | 35 |  |  |
|  |  |  |  |  |  |  |  |  |
| Asterope | 1871 | 99 | 173 | rook | 60 | 10 | 40 birds drowned in the storm | Farr 1871; The Star 1871a |
|  |  |  |  |  |  |  |  |  |
| Caduceus | 1871 | 97 | 350 | yellowhammer | 250 | 128 |  | Daily Southern Cross 1871a,b |
|  |  |  |  |  |  |  |  |  |
| Robert Henderson | 1871 | 105 | 222 | starling | 19 | 0 | imcompetency of man in charge, when he was ill | The Star 1871b |
|  |  |  |  |  |  |  |  |  |
| Santiago | 1870 | 110 | 250 | rook | 25 | 2 | did not received proper attendance | New Zealand Herald 1871a |
|  |  |  |  |  |  |  |  |  |
| Warrior Queen | 1870 | 122 | 301 | goldfinch | 60 | 31 | spoiled water | Otago Daily Times 1871; Evening Star 1871 |
|  |  |  |  | reed bunting | 4 | 4 |  |  |
|  |  |  |  | brambling | 2 | 0 |  |  |
|  |  |  |  | thrush | 60 | 42 |  |  |
|  |  |  |  | blackbird | 96 | 70 |  |  |
|  |  |  |  | dunnock | 150 | 80 |  |  |
|  |  |  |  | twite | 50 | 38 |  |  |
|  |  |  |  | redpoll | 50 | 11 |  |  |
|  |  |  |  | chaffinch | 72 | 66 |  |  |
|  |  |  |  | nightingale | 1 | 0 |  |  |
|  |  |  |  | yellowhammer | 50 | 31 |  |  |
|  |  |  |  | robin | 72 | 2 |  |  |
|  |  |  |  |  |  |  |  |  |
| Caduceus | 1871 | 112 | 286 | goldfinch | 100 | 31 |  | New Zealand Herald 1871b; Daily Southern Cross 1872a |
|  |  |  |  | dunnock | 36 | 5 |  |  |
|  |  |  |  |  |  |  |  |  |
| Charlotte Gladstone | 1871 | 90 | 308 | jackdaw | 8 | 5 | moulting in tropics | Press 1872; Farr 1872 |
|  |  |  |  | rook | 8 | 4 |  |  |
|  |  |  |  | linnet | 144 | 14 |  |  |
|  |  |  |  | redpoll | 240 | 50 |  |  |
|  |  |  |  | blackbird | 154 | 95 |  |  |
|  |  |  |  | goldfinch | 144 | 60 |  |  |
|  |  |  |  | hedge sparrow | 24 | 19 |  |  |
|  |  |  |  |  |  |  |  |  |
| Countess of Kintore | 1872 | 100 | 143 | nightingale | 14 | 0 | not properly shipped | Daily Southern Cross 1872b |
|  |  |  |  |  |  |  |  |  |
| Caller Ou | 1875 | 109 | 47 | dunnock | 76 | 14 |  | Daily Southern Cross 1875c |
|  |  |  |  |  |  |  |  |  |
| Glenlora | 1874 | 101 | 269 | dunnock | 80 | 19 | the birds were in captains cabin | New Zealand Herald 1874; Daily Southern Cross 1875a,b |
|  |  |  |  | nightingale | 12 | 0 |  |  |
| Tintern Abbey | 1875 | 116 | 7 | blackbird | 117 | 117 | wet conditions - for starlings - the worst position | Press 1875 |
|  |  |  |  | goldfinch | 110 | 110 |  |  |
|  |  |  |  | dunnock | 140 | 11 |  |  |
|  |  |  |  | linnet | 100 | 95 |  |  |
|  |  |  |  | redpoll | 120 | 120 |  |  |
|  |  |  |  | thrush | 83 | 74 |  |  |
|  |  |  |  | starling | 100 | 33 |  |  |
|  |  |  |  | yellowhammer | 180 | 180 |  |  |
|  |  |  |  |  |  |  |  |  |
| Waimate | 1879 | 90 | 330 | siskin | 65 | 52 | excessive heat and limited ventilation | The Star 1880a,b |
|  |  |  |  | thrush | 100 | 96 |  |  |
|  |  |  |  | twite | 80 | 21 |  |  |
|  |  |  |  | brambling | 110 | 107 |  |  |
|  |  |  |  | crossbill | 20 | 12 |  |  |
|  |  |  |  | redpoll | 30 | 22 |  |  |

**References**

Auckland Star 1870. Acclimatisation. Auckland Star I(128), 7 June, Page 2, <https://paperspast.natlib.govt.nz/newspapers/AS18700607.2.10>

Carrick 1870. Untitled letter to J.A. Ewen (agent in London) from 26 March. In Letter Book, 1867–1878, Otago Acclimatisation Society, 93-023/54, Hocken Collections, Dunedin, New Zealand

Colonist 1859. The Provinces. Colonist II(18), 9 August, Page 3, <https://paperspast.natlib.govt.nz/newspapers/TC18590809.2.16>

Colonist 1863. Arrival of English song birds in Nelson. Colonist VI(639), 11 December, Page 5, <https://paperspast.natlib.govt.nz/newspapers/TC18631211.2.31>

Colonist 1868. Untitled. Colonist XII(1166), 27 November, Page 2, <https://paperspast.natlib.govt.nz/newspapers/TC18681127.2.8>

Daily Southern Cross 1862. Progress of acclimatisation. Daily Southern Cross XVIII(1502), 9 May, Page 9, <https://paperspast.natlib.govt.nz/newspapers/DSC18620509.2.38>

Daily Southern Cross 1867a. Auckland Acclimatisation Society. Daily Southern Cross XXIII(3208), 28 October, Page 3, <https://paperspast.natlib.govt.nz/newspapers/DSC18671028.2.13>

Daily Southern Cross 1867b. Arrival of the ship Water nymph, from London. Daily Southern Cross XXXIII(3238), 2 December, Page 8, <https://paperspast.natlib.govt.nz/newspapers/DSC18671202.2.39.1>

Daily Southern Cross 1869. Meeting of the acclimatisation society. Daily Southern Cross XXV(3577), 5 January, Page 4, <https://paperspast.natlib.govt.nz/newspapers/DSC18690105.2.29>

Daily Southern Cross 1870a. Acclimatisation Society. Daily Southern Cross XXVI(3937), 5 April, Page 4, <https://paperspast.natlib.govt.nz/newspapers/DSC18700405.2.25>

Daily Southern Cross 1870b. Rooks. Daily Southern Cross XXVI(4070), 7 September, Page 3, <https://paperspast.natlib.govt.nz/newspapers/DSC18700907.2.18>

Daily Southern Cross 1870c. Importation of rooks. Daily Southern Cross XXVI(4096), 6 October, Page 4, <https://paperspast.natlib.govt.nz/newspapers/DSC18701006.2.22>

Daily Southern Cross 1871a. Auckland Acclimatisation Society. Monthly meeting of council. Daily Southern Cross XXVII(4231), 7 March, Page 3, <https://paperspast.natlib.govt.nz/newspapers/DSC18710307.2.14.3>

Daily Southern Cross 1871b. Auckland Acclimatisation Society. Monthly meeting. Daily Southern Cross XXVII(4255), 4 April, Page 3, <https://paperspast.natlib.govt.nz/newspapers/DSC18710404.2.21>

Daily Southern Cross 1872a. Shipping summary. Daily Southern Cross XXVIII(4521), 21 February, Page 2, <https://paperspast.natlib.govt.nz/newspapers/DSC18720221.2.3>

Daily Southern Cross 1872b. Acclimatisation society. Daily Southern Cross XXVIII(4689), 3 September, Page 3, <https://paperspast.natlib.govt.nz/newspapers/DSC18720903.2.20>

Daily Southern Cross 1875a. Untitled. Daily Southern Cross XXXI(5420), 6 January, Page 2, <https://paperspast.natlib.govt.nz/newspapers/DSC18750106.2.9>

Daily Southern Cross 1875b. Acclimatisation society. Daily Southern Cross XXXI(5431), 19 January, Page 3, <https://paperspast.natlib.govt.nz/newspapers/DSC18750119.2.18>

Daily Southern Cross 1875c. Miscellaneous. Daily Southern Cross XXXI(5553), 10 June, Page 7, <https://paperspast.natlib.govt.nz/newspapers/DSC18750610.2.48>

Evening Star 1871. Acclimatisation society. Evening Star VIII(2509), 2 March, Page 2, <https://paperspast.natlib.govt.nz/newspapers/ESD18710302.2.9>

Farr, S. 1871.Untitled letter to T. Nottidge from 30 September. In Letter Books (1869 – 1884), Canterbury Acclimatisation Society, ANZC Archives (ARCHIVE 5 1), Christchurch City Library. <https://christchurch.bibliocommons.com/item/show/235416037>

Farr, S. 1872. Untitled letter to A. O. Ottywell from 15 February. In Letter Books (1869 – 1884), Canterbury Acclimatisation Society, ANZC Archives (ARCHIVE 5 1), Christchurch City Library. <https://christchurch.bibliocommons.com/item/show/235416037>

Hawke's Bay Herald 1865. Importation of birds. Hawke's Bay Herald 8(682), 12 September 1865, <https://paperspast.natlib.govt.nz/newspapers/HBH18650912.2.13>

Hawke's Bay Herald 1869. Acclimatisation. Hawke's Bay Herald 13(1016), 23 January, Page 3, <https://paperspast.natlib.govt.nz/newspapers/HBH18690123.2.22.3>

King, E. 1863. Untitled letter. Daily Southern Cross XIX(1839), 9 June, Page 3, <https://paperspast.natlib.govt.nz/newspapers/DSC18630609.2.13.2>

Lyttelton Times 1864. The birds by the British Empire. Lyttelton Times XXII(1289), 10 September, Page 5, <https://paperspast.natlib.govt.nz/newspapers/LT18640910.2.23>

Lyttelton Times 1867. Canterbury Acclimatization Society. Lyttelton Times XXVII(1904), 26 January, Page 2, <https://paperspast.natlib.govt.nz/newspapers/LT18670126.2.11>

Morrin, W. 1863. Untitled letter. Daily Southern Cross XIX(1839), 9 June, Page 3, <https://paperspast.natlib.govt.nz/newspapers/DSC18630609.2.13.2>

Nelson Evening Mail 1869. Importation of birds. Nelson Evening Mail IV(93), 22 April, Page 2, <https://paperspast.natlib.govt.nz/newspapers/NEM18690422.2.7>

Nelson Examiner and New Zealand Chronicle 1865. Acclimatization. Nelson Examiner and New Zealand Chronicle XXIV(36), 12 September, Page 2, <https://paperspast.natlib.govt.nz/newspapers/NENZC18650325.2.9>

Nelson Examiner and New Zealand Chronicle 1868. Importation of birds. Nelson Examiner and New Zealand Chronicle XXVII(80), 4 July, Page 6, <https://paperspast.natlib.govt.nz/newspapers/NENZC18680704.2.31.5>

New Zealand Herald 1864. English birds imported. New Zealand Herald I(212), 18 July, Page 3, <https://paperspast.natlib.govt.nz/newspapers/NZH18640718.2.16>

New Zealand Herald 1869a. Acclimatisation at Wellington. New Zealand Herald VI(1603), 14 January, Page 4, <https://paperspast.natlib.govt.nz/newspapers/NZH18690114.2.17>

New Zealand Herald 1869b. Acclimatisation. Importation of English birds. New Zealand Herald VI(1637), 19 February, Page 3, <https://paperspast.natlib.govt.nz/newspapers/NZH18690219.2.26>

New Zealand Herald 1869c. Acclimatisation. New Zealand Herald VII(1859), 31 December, Page 4, <https://paperspast.natlib.govt.nz/newspapers/NZH18691231.2.19>

New Zealand Herald 1870a. The City of Auckland. New Zealand Herald VII(1885), 31 January, Page 5, <https://paperspast.natlib.govt.nz/newspapers/NZH18700131.2.25>

New Zealand Herald 1870b. Untitled. New Zealand Herald VII(1985), 30 May, Page 3, <https://paperspast.natlib.govt.nz/newspapers/NZH18700530.2.12>

New Zealand Herald 1870c. Acclimatization. New Zealand Herald VII(2016), 6 July, Page 6, <https://paperspast.natlib.govt.nz/newspapers/NZH18700706.2.34>

New Zealand Herald 1871a. Acclimatization society. New Zealand Herald VIII(2171), 10 January, Page 2, <https://paperspast.natlib.govt.nz/newspapers/NZH18710110.2.14>

New Zealand Herald 1871b. Acclimatisation society. New Zealand Herald, VIII(2459), 12 December, Page , <https://paperspast.natlib.govt.nz/newspapers/NZH18711212.2.22>

New Zealand Herald 1874. Untitled. New Zealand Herald XI(4073), 2 December, Page 2, <https://paperspast.natlib.govt.nz/newspapers/NZH18741202.2.12>

New Zealander 1860a. Untitled. New Zealander XVI(1508), 29 September, Page 2, <https://paperspast.natlib.govt.nz/newspapers/NZ18600929.2.15>

New Zealander 1860b. New arrivals and fresh blood. New Zealander XVI(1521), 14 November, Page 3, <https://paperspast.natlib.govt.nz/newspapers/NZ18601114.2.12>

Oamaru Times 1867. Untitled. Oamaru Tmes IX(272), 31 December, Page 2, <https://paperspast.natlib.govt.nz/newspapers/NOT18671231.2.6>

Oamaru Times 1870. General summary. Oamaru Times XIII(494), 18 February, Page 2, <https://paperspast.natlib.govt.nz/newspapers/NOT18700218.2.7>

Otago Acclimatisation Society (unpublished). Otago Acclimatisation Society Minute Book, 1871–1891. MS378/B, Hocken Collections, Dunedin, New Zealand

Otago Daily Times 1871. Acclimatisation. Otago Daily Times, Issue 2826, 27 February, Page 2, <https://paperspast.natlib.govt.nz/newspapers/ODT18710227.2.15>

Otago Witness 1864. News of the week. Otago Witness, Issue 660, 23 July, Page 13, <https://paperspast.natlib.govt.nz/newspapers/OW18640723.2.32>

Press 1866. Acclimatization. Press X (1291), 27 December, Page 2, <https://paperspast.natlib.govt.nz/newspapers/CHP18661227.2.9>

Press 1872. Acclimatisation society. Press XIX (2727), 27 January, Page 2, <https://paperspast.natlib.govt.nz/newspapers/CHP18720127.2.14>

Press 1875. Acclimatisation. Press XXIII(3027), 5 May, Page 2, <https://paperspast.natlib.govt.nz/newspapers/CHP18750505.2.14>

Taranaki Herald 1866. Untitled. Taranaki Herald XIV(701), 6 January, Page 3, <https://paperspast.natlib.govt.nz/newspapers/TH18660106.2.11>

The Star 1871a. Acclimatisation society. The Star, Issue 1130, 30 September, Page 2, <https://paperspast.natlib.govt.nz/newspapers/TS18710930.2.6>

The Star 1871b. Acclimatisation. The Star, Issue 1176, 24 November, Page 2, <https://paperspast.natlib.govt.nz/newspapers/TS18711124.2.4>

The Star 1880a. Shipment of English birds. The Star, Issue 3711, 15 January, Page 2, <https://paperspast.natlib.govt.nz/newspapers/TS18800115.2.8>

The Star 1880b. Acclimatisation society. The Star, Issue 3711, 5 March, Page 2, <https://paperspast.natlib.govt.nz/newspapers/TS18800305.2.10>

Wellington Independent 1865. Birds. Wellington Independent XX(2205), 13 May, Page 5, <https://paperspast.natlib.govt.nz/newspapers/WI18650513.2.19>

Wellington Independent 1868. Acclimatisation. Wellington Independent XXII(2633), 8 February, Page 4, <https://paperspast.natlib.govt.nz/newspapers/WI18680208.2.15>
