# Supplementary material for: Lasting the distance: The survival of alien birds shipped to New Zealand in the 19th century
Source: Ecol Evol. 2020 Mar 7;10(9):3944–53. doi: 10.1002/ece3.6143 (PMC7244811; doi:10.1002/ece3.6143)
Supplement: Supplementary file 4 [file ECE3-10-3944-s004.docx]

**Supplementary table 4.** Likelihood ratio test contrasting models, i.e. generalized linear mixed models (with beta-binomial logit function) with proportion of surviving bird individuals out of the total loaded as a response variable, containing the binary classification of voyages where Richard Bills (or his son Henry) cared for the birds versus not. Difference in model deviance is assumed to be distributed as *χ*^2^ with specified degrees of freedom (*df*).

| Model comparison | Number of parameters | Log-likelihood | Deviance | *df* | *P*-value |
| --- | --- | --- | --- | --- | --- |
| Intercept only | 4 | -240.37 | 5.83 | 1 | 0.016 |
| Bills on board | 5 | -237.46 |  |  |  |
